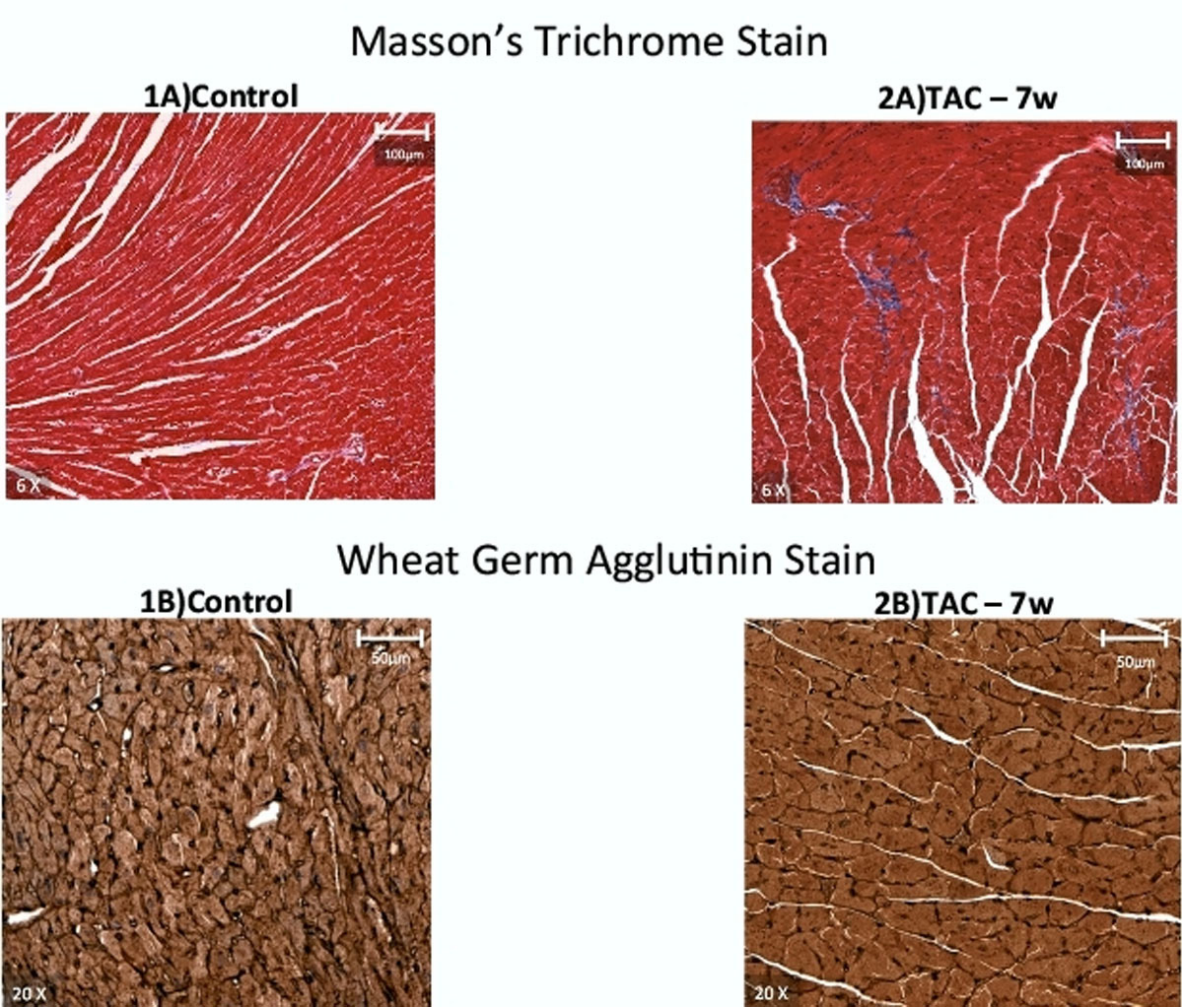# Cellular hypertrophy occurs before interstitial fibrosis in pressure-overload heart failure

**DOI:** 10.1186/1532-429X-15-S1-O2

**Published:** 2013-01-30

**Authors:** Ravi Shah, Otavio R Coelho-Filho, Richard N Mitchell, Raymond Y Kwong, Saumya Das, Anthony Rosenzweig, Michael Jerosch-Herold

**Affiliations:** 1 Brigham and Women's Hospital, Boston, MA, USA; 2 Department of Internal Medicine, State University of Campinas (UNICAMP), Sao Paulo, Brazil; 3 Beth Israel Deaconness Medical Center, Boston, MA, USA

## Background

Hypertension is a major risk factor for diastolic heart failure. Hypertension causes cardiac hypertrophy through both individual cardiomyocyte hypertrophy as well expansion of the extracellular matrix. Methods to independently quantify cellular size and fibrosis will improve definition of the pathophysiology of heart failure and opportunities for early intervention.

## Methods

11 mice underwent transverse aortic constriction (TAC). All mice were imaged at baseline, and a group of mice were examined early (2 weeks), intermediate (4 weeks) and late (7 weeks) post-TAC with histologic correlation. Mice were imaged at 9.8 Tesla with standard cine function sequences and T1 quantification via a Look-Locker cine experiment, pre and post gadolinium. The intra-cellular lifetime of water (τic), a-cell size dependent parameter, and the extracellular volume fraction (ECV) were determined by fitting R1 in tissue, against R1 in the blood pool, with a 2-site model for transcytolemmal water exchange. Minor (Dmin) and major (Dmaj) cell-diameters were determined by image analysis of FITC-wheat germ agglutinin stained sections.

## Results

TAC induced significant and sustained LV hypertrophy (96.7±16.7 mg at baseline controls vs. 120.491±11.702mg at 2 weeks, P<0.05; 146.755±8.167mg at 4 weeks, P<0.05 vs. baseline controls, 147.767±8.155 at 7 weeks, P<0.05 vs. baseline controls). TAC was also associated with a significant decrease in LVEF (all P<0.005 vs. baseline, Table 2). Despite a significant increase in LV mass, mice exposed to TAC did not have as significant an increase in collagen volume fraction or myocardial ECV by CMR at 2 weeks, 4 weeks, or 7 weeks after TAC.

TAC mice showed evidence of increased cardiomyocyte size and volume by cellular dimension at every time point post-TAC (P=0.049 across time points). TAC mice had progressive increases in τic depending on length of exposure to TAC (0.218±0.049 at 2 weeks, 0.255±0.031 at 4 weeks, and 0.488±0.180 at 7 weeks, P=0.048). τic was associated with cellular surface to volume ratio (r = 0.84, p = 0.001).

## Conclusions

Cellular hypertrophy occurs before interstitial fibrosis in mice exposed to pressure-overload. These results suggest that CMR identifies an early, potentially reversible cellular phenotype in diastolic or hypertensive heart failure.

## Funding

Drs. Coelho-Filho and Shah are supported by Post-Doctoral Fellowships from the American Heart Association (AHA 11POST5550053 to OCF and AHA 11POST110033 to RVS). Dr. Kwong is supported in part by a research grant from the National Institutes of Health (NIH RO1 HL 091157). Dr. Jerosch-Herold is supported in part by a research grant from the National Institutes of Health (1R01HL090634-01A1).

**Figure 1:**